# The Rise of *Candida auris* in Mauritius

**DOI:** 10.1093/ofid/ofae696

**Published:** 2024-11-25

**Authors:** Dooshanveer C Nuckchady

**Affiliations:** Department of Internal Medicine, Victoria Hospital, Candos, Quatre Bornes, Mauritius

To the  Editor—I read with interest the article by Dr Schaefer and coauthors regarding a rise in *Candida auris* in New York City [1]. Their article also reflects what has been noticed in the Republic of Mauritius. After the Central Health Laboratory of the country detected the first cases of *C auris* in February 2021 by using matrix-assisted laser desorption/ionization time-of-flight mass spectrometry, the number of clinical cases continued to rise, especially in the intensive care units (ICUs) of the island (see Figure 1 for details).

**Figure 1.**
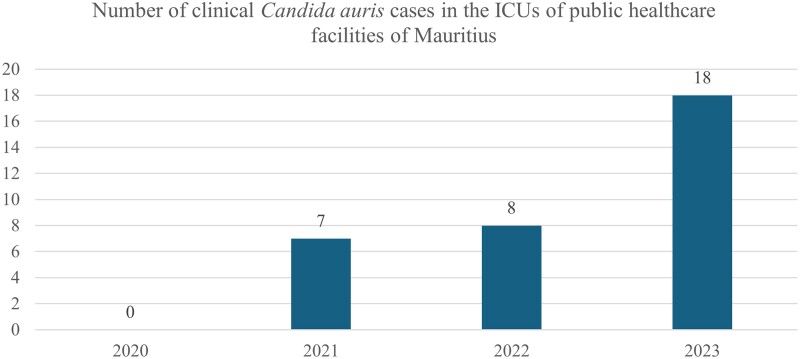
Clinical cases of *Candida auris* have continued to rise in the intensive care units (ICUs) of Mauritius since 2021. This bar chart displays data obtained from 9 ICUs, with a total bed count of about 70, in 6 hospitals of the public health care sector.

Schaefer et al propose the following reasons for such a rise during the COVID-19 pandemic: extended use of personal protective equipment (PPE), abuse of antimicrobials, an increase in colonization with other multidrug-resistant organisms, breaches in hand hygiene, inadequate cleaning or disinfection, a rise in mechanical ventilation, and a scarcity of staff [1].

Other authors have suggested that climate change may also play a role, given that *C auris* prefers a warmer temperature to grow [2]. Interestingly, quaternary ammonium compounds have no or poor activity against *C auris* [3]. Use of these compounds increased in health care institutions in Mauritius from 2020 to 2022, while hospitals were noted to be using hypochlorite solution that was too dilute. In addition, exposure to carbapenems and azoles has been associated with an upsurge in *C auris* [4].

In 2021, the Ministry of Health and Wellness of Mauritius listed *C auris* as one of its high-priority multidrug-resistant organisms that require surveillance under the NOHARM system: National One Health Antimicrobial Resistance Monitoring. By using a systematic process, outbreaks are regularly notified to public health care facilities for actions to be taken—an outbreak of *C auris* is currently defined in Mauritius as at least 2 cases occurring in the same week in the same ward. All patients who are infected with this organism are expected to be isolated under strict contact precautions. However, poor compliance has been noted with this directive in multiple health care institutions.

In a report from the Ministry of Health and Wellness, the most common risk factors linked to health care workers acquiring COVID-19 in Mauritius were poor ventilation at the site of work and incorrect use of PPE [5]. The latter, especially the reuse of disposable gowns and gloves across multiple patients, could have also led to the spread of *C auris*. Moreover, compliance to hand hygiene dropped from 12% to 1% in 2021 [6]. Inadequate cleaning and disinfection of ventilators and their circuits were already a known problem before the outbreak of *C auris* started in Mauritius; specifically, after disinfection, 60% of ventilators (6 of 10) still tested positive for multidrug-resistant organisms in 2020 in 1 ICU [7].

Since several countries globally are now describing the emergence of *C auris*, including a neighboring island in the Indian Ocean called La Réunion [8], additional data should be collected to improve our understanding of how transmission occurs to help stop patients from being infected. Measures that can be taken include using disinfectants with fungicidal properties; adhering to basic principles of infection prevention and control, such as hand hygiene and PPE compliance; screening patients who are admitted to ICUs; and identifying environmental reservoirs of *C auris* through microbiological investigations.

## Notes

***Acknowledgments***. The Central Health Laboratory of Candos (Mauritius) cultured and identified the organisms.
